# Determinants of caries experience and the impact on the OHRQOL of 6-year-old Libyan children: a cross-sectional survey

**DOI:** 10.1186/s12903-021-01681-2

**Published:** 2021-06-25

**Authors:** Lamis Ballo, Arheiam Arheiam, Jamaludin Marhazlinda

**Affiliations:** 1 Department of Community Oral Health and Clinical Prevention, Faculty of Dentistry, University of Malaya, Kuala Lumpur, Malaysia; 2 Department of Community and Preventive Dentistry, Faculty of Dentistry, University of Benghazi, Benghazi, Libya

**Keywords:** Child, Oral health related quality of life, Libyan children, A-ECOHIS

## Abstract

**Objective:**

The current study aimed to assess the caries experience and associated factors and its impact on the oral health-related quality of life (OHRQoL) among 6-year-old Libyan children.

**Methods:**

A cross-sectional survey including 706 six-year-old children was conducted in 2017 in Benghazi, Libya. Data were collected through a self-administered questionnaire assessing socioeconomic status and oral health behaviours, and the Arabic version of the Early Childhood Oral Health Impact Scale (A-ECOHIS) to assess the OHRQoL. Clinical examination assessed caries experience at tooth level (dmft) and the number of decayed, missing due to caries and filled teeth (dt, mt and ft). Poisson regression analysis was performed to determine the association between dmft scores and the independent predictors. Linear regression analysis was conducted for ECOHIS scores with the children’s gender, SES and OHB. The statistical significance was set to ≤  0.05.

**Results:**

Data were available for 706 children. Caries prevalence (dt) and dmft of ≥ 1 were 69.1% and 71% respectively. The mean ± SD dmft score was 3.23 ± 3.32. There was a significant and direct association between dmft scores and daily consumption of sugary snacks (B = 1.27, P = 0.011) and a significant inverse association with teethbrushing twice daily (B = 0.80, P = 0.041). There was a significant and direct association between A-ECOHIS and dmft (B = 1.14, P ≤ 0.001) and a significant and inverse association between A- ECOHIS and high and intermediate family income compared to low income (B = −3.82, P = 0.0001 and B = −2.06, P = 0.028).

**Conclusions:**

6-year-old Libyan children had a relatively high caries experience an untreated decay with impact on OHRQoL. Social disparities, sugar consumption patterns and oral hygiene practices were associated with high caries experience.

## Introduction

Dental caries is among the most prevalent health conditions of childhood [1], and characterised by wide socioeconomic disparities within and between countries [2–5]. Although prevalence and severity of caries in young children have declined in developed countries [6], this is not the case in many developing countries and among underprivileged groups [7–9]. Untreated caries can have significant impacts on the OHRQoL and wellbeing of children and their parents in the short and long terms [10]. Caries is associated with tooth pain, discomfort, and difficulties in sleeping and eating [11, 12], which affect children’s daily performance at the school [13], social activities and self-esteem [14]. In addition, caries management can be very expensive with substantial costs and financial burdens on families [15]. Yet, caries is a preventable disease provided that its risk factors identified and eliminated.

Caries is a complex process that is initiated by acids produced by bacterial metabolism of fermentable carbohydrates [11], and influenced by a range of biological and behavioural, social and wider contextual factors [11, 16]. For instance, caries development and progression is affected by individual’s behaviours such as sugar consumption, oral hygiene practices and dental attendance as well as social and wider contextual factors such as socioeconomic status [17–19], women’s empowerment and participation in decision making in the community [20] and political status in the country [21]. This may explain the worldwide variations in caries distribution and aetiology [19, 22, 23]. Therefore, identifying caries associated factors and high-risk groups in their context is crucial to tailor effective and efficient caries preventive strategies.

There is a paucity of research that investigated caries, its impacts and associated factors among young children in Africa [3, 24]. Libya, one of the Arabic league countries which is located on the North coast of Africa, is no exception. The country is a war-torn and endured several civil wars and fiscal crises since what is so-called February uprising in 2011. Such an environment of political unrest and fiscal hardships has been linked to decreased availability of sugars and eventually decreased caries incidence [21]. A recently published natural experiment conducted among Libyan children has confirmed this view and demonstrated a concomitant decrease in sugar availability and caries experience in permanent teeth of 12-year-olds [25]. However, little is known about early childhood caries and its related risk factors in primary teeth of Libyan children. A search of the literature revealed few studies conducted in Libya, which did not investigate caries risk factors [26]. To fill this knowledge gap, the present study aims to investigate caries experience, OHRQoL impacts and its associated factors among 6-year-old Libyan children. The study investigated following null hypotheses: (1) There is no association between dental caries and sociodemographic and behavioural variables. (2) OHRQoL has no association with sociodemographic variables and caries experience.

## Methods

Ethical clearance and permissions to conduct the study were secured from the Libyan Ministry of Health and local authorities in Benghazi. Ethics approval was obtained from a local research ethics committee (Ref: 17/LB/1005). The study methods performed entirely in accordance with the relevant guidelines and regulations of the World Medical Association Declaration of Helsinki. Informed consent was obtained from the parents⁄guardians before commencing the study.

A cross-sectional survey was conducted in the city of Benghazi, the second-largest city in the country and the melting pot of Libyan citizens since its inhabitants descend from various Libyan tribes and cities. The study population included all Libyan children who will reach their 6^th^ birthday on November and December 2017. The study sample was recruited from preschool children, attending an obligatory vaccination for primary school starters and therefore an uptake higher than 95% was expected. The vaccination was conducted at 20 health centres in Benghazi, between August and September 2017. Each health centre was responsible for the vaccination of children in their local area. The health centres are distributed over the three main administrative districts of the city of Benghazi to cover equal numbers of children in each local area. Therefore, the health centres were used as sampling points to randomly select study sample.

**Fig. 1:**
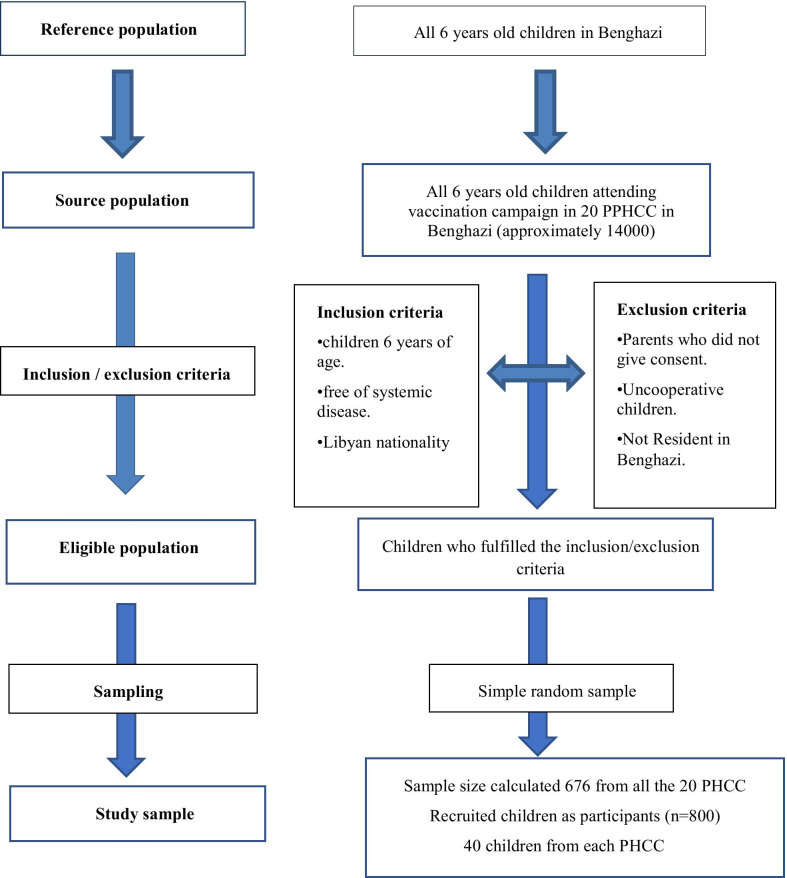
flowchart of sampling

It was estimated that 14000 6-year-old children with almost equal gender distribution, were targeted for vaccination in Benghazi in 2017. Sample size calculations were based on the finding of a pilot study showing that 55% of preschool children had dental caries. Given this, a minimal sample size of 676 children was identified as sufficient to allow an estimate of the proportion of children who have caries experience (dmft > 0) with 95% confidence level and 0.05% error margin. The sample size was increased to 800 children to compensate for potential non-response. Therefore, 40 children were targeted and randomly selected from each sampling point (20 health centres). If parents did not agree to participate, the next child in the list was selected. Inclusion criteria were the Libyan nationality and reaching 6 years of age according to the child’s last birthday. Exclusion criteria included major systemic illnesses, disabilities, and failure to obtain consent to take part in the study (Fig. 1).

Data were collected through a self-administered questionnaire and clinical oral examinations. The selected participants were given the study’s information leaflet and asked to complete a structured questionnaire at home. An appointment for clinical oral examination was then arranged at the Paediatric Dentistry clinics of the University of Benghazi. Attending the examination was implied as a consent to take part in the study. On arrival to the examination appointment, the principal investigator (LB) reviewed the questionnaire with parents for completeness and clarification. The questionnaire was developed from previous studies [19] and pretested for clarity and face validity among a group of parents attending the Faculty of Dentistry clinics. The questionnaire comprised of close-ended questions and takes, on average, 20 minutes to be completed. The questionnaire included 2 parts: (1) demographic data (gender) and socioeconomic data (mothers’ education and family income); and (2) oral health behaviours (tooth brushing and sugar snacking habits). The parents/caregivers were also asked to complete the Arabic version of the Early Childhood Oral Health Impact Scale (A-ECOHIS), previously tested for validity and reliability in Arabic speaking population [27]. The original ECOHIS has two sections: children section and family section, which, respectively, test the OHRQoL impacts on children and their families [28]. The A-ECOHIS It consists of 13 items within two main parts: the child impact section consists of four items and the family impact section consists of nine items distributed over subdomains as follows: (1) Child subdomains: symptoms, function, psychology and self-image; (2) Family subdomains: distress and parental function). Responses are on a five-point scale (0 = never to 4 = very often). ECOHIS scores are obtained by summing responses for all 13 questions. The child impact section range of score is 0 to 36 and the score range for family impact section is from 0 to 16. The total score ranges between 0 and 52, and the higher ECOHIS score means poor OHRQoL and/or a great impact of OHRQoL.

Clinical oral examinations were carried out by single calibrated examiner (LB) in the presence of parents or caregivers. All examinations were carried out visually under artificial light using disposable dental mirrors only, while the child seated on a dental chair. Before clinical examinations, tooth surfaces were cleaned with wet gauze pads to remove any loose debris. Dental caries was then assessed and recorded according to the World Health Organisation diagnostic criteria [29]. Caries prevalence was measured as the percentage of population affected by dental caries and caries severity was measured using dmft indices which were calculated as the sum of decayed, missing and filled teeth. During data collection, a subgroup of 20 children was randomly selected for duplicate examinations, to assess intra-examiner agreement. Kappa value of 0.8 was achieved which indicated good agreement.

The Statistical Package for Social Sciences, version 24 (SPSS Inc., Chicago, IL, USA) was used for data management and analysis. Descriptive statistics were used for sample characteristics, caries prevalence and severity and A-ECOHIS scores. Mann Whitney U test and Kruskal Wallis test were used to compare A-ECOHIS scores and caries experience. Poisson regression models were fitted for dmft score as an outcome variable. The Poisson regression model was adjusted for mothers’ education, family income, child’s gender, tooth brushing frequency, between meals snacking and near bedtime consumption of sugary drinks. Maternal education was classified as (1) primary, (2) High school, (3) tertiary, while family income was categorised as (1) low (less than 500 Libyan Dinar), (2) intermediate (500–1500 Libyan Dinar), (3) high (above 1500 Libyan Dinar). The family income categories were based on current classification of income according to the social security fund in Libya. Linear regression analysis was conducted for ECOHIS score children’s gender, family income, mothers’ education and dmf scores were the explanatory variables. The statistical significance for all statistical procedures was set at ≤ 0.05.

## Results

Out of 800 children recruited to take part in the study, 706 fulfilled the eligibility criteria and included in the final analysis, giving a response rate of 88 %. Table 1 shows the demographic and socioeconomic characteristics of the study sample. The gender distribution was almost equal. Most of the mothers (45%) had tertiary education, whereas most of the families have an intermediate income (76.6%). Near bedtime intake of sugary drinks was relatively low (18%), but in-between meals intake of sugary snacks was quite common (48.6%). On the other hand, most of the parents (81.5%) reported that their children brush their teeth.

**Table 1:** Sociodemographic characteristics and oral health behaviours of study participants (n = 706)

Variable	Frequency	%
*Child gender*		
Male	361	51.1
Female	345	48.9
*Mother’s educational level*		
Primary	145	20.5
High school	243	34.4
Tertiary	318	45.1
*Family income*		
Low	64	9.1
Intermediate	541	76.6
High	101	14.3
*Teeth brushing frequency*		
No brushing	131	18.5
Brush irregularly	196	27.8
Brush regularly once	218	30.9
Brush regularly twice or more	161	22.8
*Sugary drinks bedtime*		
Not daily	579	82.0
Once/ day	127	18.0
*Sweet Snacks between meals*		
Not daily	117	16.6
Once /day	246	34.8
2–3 /day	343	48.6

Table 2 presents the caries distribution of the study sample. Overall, 502 out of 706 children (71.1%) had dmft ≥ 1. The mean scores of dmft was (3.23, SD = 3.32). Whilst most of the children had untreated dental caries (69.1%), small proportions of them had filled (9.1%) or missing teeth (8.1%). Likewise, the mean numbers of decayed teeth (2.92, SD = 3.15) are higher than that for missing or filled ones.

**Table 2:** Dental caries experience of study participants (N = 706)

Variables of study	Mean (SD)	Freq (%)
Decayed teeth (dt)	2.92 (3.15)	488 (69.1%)
Missing teeth (mt)	0.14 (0.55)	57 (8.1%)
Filled teeth (ft)	0.18 (0.69)	64 (9.1%)
dmft	3.23 (3.32)	502 (71.1%)

Table 3 shows bivariate analysis and Poisson regression models for dmft as an outcome variable. Statistically significant findings were observed in association with dietary habits and oral hygiene habits. On one hand, regular tooth brushing twice a day was associated with lower scores of dmft (B = 0.80, P = 0.41)On the other hand, daily intake of near bedtime sugary drinks was associated with higher dmft scores compared to the reference groups (B = 1.27, P = 0.11)..

**Table 3:** Associations between dental caries in deciduous teeth at 6 years of age and sociodemographic and behavioural variables

Variable		Mean (SD)	P value	Adjusted Cof. (95% CI)	P value
*Gender*					
Male		3.2 (3.4)	0.476	Reference	–
Female		3.2 (3.2)		0.97(0.81, 1.13)	0.655
*Mother education*					
Primary		3.5 (3.3)	0.254	Reference	–
High school		3.2 (3.4)		0.95 (0.77, 1.18)	0.668
Tertiary		3.1 (3.4)		0.93 (0.76, 1.15)	0.530
*Family income*					
Low		3.7 (4.2)	0.454	Reference	–
Intermediate		3.2 (3.3)		0.91 (0.66, 1.25)	0.544
High		2.7 (2.9)		0.78 (0.49, 1.24)	0.294
*Teeth brushing frequency*					
Never/irregularly brush		3.4 (3.5)	0.162	Reference	–
Brush regularly once		3.3 (3.3)		0.98 (0.82, 1.18)	0.847
Brush regularly twice		2.7 (2.8)		0.80 (0.65, 0.99)	0.041*
*Sugary drinks bedtime*					
Never/sometimes		3.1 (3.2)	0.012*	Reference	–
Daily		4.0 (3.8)		1.27 (1.06, 1.54)	0.011*
*Sweet snacks between meals*					
Not daily		3.7 (3.7)	0.126	Reference	–
Once/ day		2.9 (3.2)		0.80 (0.64, 1.01)	0.059
2_3/ day		3.3 (3.2)		0.88 (0.71, 1.09)	0.237

*P value < 0.05 poison models were adjusted for children gender, parents’ education, family income, tooth brushing frequency, sugar intake frequency and near bedtime consumption

Table 4 shows the distribution of A-ECOHIS total scores and its sections (child and family) and subdomains. The reported range of overall ECOHIS score was 0–39. The mean scores were [5.37 (SD = 6.58), 4.13(SD = 4.87), 1.24 (SD = 1.28)], for overall A-ECOHIS, and child and family sections, respectively. A-ECOHIS score ≥ 1) was 70.9% for the overall impact, 70.6% for children and 34% for family impact. The highest impact scores were observed in symptoms and function domains in child section, followed by parental distress in family section. Comparison of these scores according to caries experience (dmft = 0 vs. dmft ≥ 1) demonstrated higher impact on OHRQoL among children who had caries experience, in overall scores of A-ECOHIS and its sections and domains (P ≤ 0.001).

**Table 4:** Description of A-ECOHIS scores and comparison of total and subdomain scores according to caries experience among study participants (n = 706)

Variables of study (N = 706)	Possible range	Range	Mean (SD)	Means (SD) Children had caries	Means (SD) Children had no caries	P value
A-ECOHIS (Total)	0–52	0–39	5.37(6.58)	6.98 (6.88)	3.3 (0.23)	≤ 0.001
*Child domain*	0–36	0–29	4.13 (4.87)	5.32 (5.01)	1.17 (2.87)	≤ 0.001
Symptoms	0–4	0–4	1.29 (1.10)	1.76 (0.93)	0.13 (0.44)	
Function	0–16	0–14	1.74 (0.63)	2.19 (2.68)	0.58 (1.45)	
Psychology	0–8	0–8	0.72 (0.71)	0.90 (1.52)	0.26 (0.85)	
Self-image	0–8	0–8	0.39 (0.55)	0.45 (1.16)	0.23 (0.73)	
*Family domain*	0–16	0–12	5.26 (2.33)	1.66 (2.55)	0.20 (0.77)	≤ 0.001
Parental distress	0–8	0–8	0.99 (0.94)	1.35 (2.09)	0.15 (0.60)	
Family function	0–8	0–5	0.24 (0.36)	0.32 (0.82)	0.05 (0.34)	

Mann Whitney U t test was used to compare scores of A-ECOHIS and its subsection according to caries experience (dmft = 0 vs dmft ≥ 1)

Table 5 shows linear regression models for the association between caries experience presented as dmft score and ECOHIS scores. Statistically significant positive association (B = 1.15, P ≤ 0.001)was observed and remained significant after the adjustment for mothers’ education and family income as well as child’s gender(B = 1.14, P ≤ 0.001). Statistically significant association was also observed between ECOHIS impact and family income .The higher the family income, the lower the ECOHIS impact B = − 3.82, P = 0.001).

**Table 5:** Associations between A-ECHOIS impact in deciduous teeth at 6 years of age and sociodemographic variables and dmft score

Variable		Unadjusted Cof. (95% CI)	P value	Adjusted Cof. (95% CI)	P value
*Gender*					
Male		Reference		Reference	
Female		0.29 (−0.67, 1.27)	0.548	0.23 (−0.36, 1.81)	0.291
*Mother education*					
Primary		Reference		Reference	
High school		−0.63 (−1.98, 0.72)	0.359	−0.72 (−1.89, 0.47)	0.141
Tertiary		0.27 (−1.02, 1.55)	0.645	0.21 (−1.11, 1.47)	0.235
*Family income*					
Low		Reference		Reference	
Intermediate		0.01 (−1.47, 1.47)	0.996	−2.06 (−3.99, −0.14)	0.028 *
High		−1.76 (−3.71, 0.19)	0.076	−3.82 (−6.24, −1.41)	0.001**
*dmft*		1.15 (1.03, 1.27)	0.000*	1.14 (1.02, 1.26)	≤ 0.001***

*P value < 0.05, ** ≤ 0.01, *** ≤ 0.01. Linear regression models for A-ECHOIS scores were adjusted for children gender, mothers’ education, family income and dmft score

## Discussion

To authors’ best knowledge, this is first population survey investigating caries experience, OHRQoL impacts and its related factors among 6-year old Libyan children. The study sample was recruited from children attending a compulsory vaccination campaign. Many previous studies recruited pre-school children from nurseries which is not a reliable option in Libya because most nurseries are run by the private sector and their attendance is influenced by the financial circumstances of the families, leading eventually to a non-representative sample. Therefore, the sampling approach adopted in the present study is considered a practical and enhanced the representativeness of the sample. The primary aim of the present study was to investigate caries experience among 6-year old Libyan children. The data shows that prevalence of dental caries in primary dentition (dmft ≥ 1) was 71.1%, with an average dmft of 3.23 (SD = 3.32). These figures while comparable to that reported in some Middle Eastern and Arab league countries [24], they are higher than that reported in Africa and Europe [3]. This relatively high caries prevalence and severity is a rather surprising in the light of the ongoing political unrest in Libya, which is expectedly associated with decreased availability of sugars and decreased caries levels [21, 25]. However, it could be the case that high caries rates in our study are attributed to the sophisticated sampling and clinical examination methods which enhanced caries detection, in comparison to other epidemiological studies which used natural light source tor small convenience samples such that previously conducted in Libya [26].

In the present study most of the participants had untreated dental caries. This finding accords with that of previous studies conducted among Libyan children, showing highly unmet treatment needs [30, 31]. Possible explanations for this observation may be that the dentists lack the competence to communicate with and manage the behaviour of young children [32] or that parents underestimate the importance of deciduous teeth [33], eventually leading to either poor access or poor utilization of dental care. However, these remin assumptions and further research is required to explore parental attitudes towards oral health of their children and the provision as well as uptake of dental services in Libya, to fully understand the phenomenon of highly unmet treatment needs among Libyan children.

The current study investigated social and behavioural risk factors related to caries in 6-year olds. Our study confirms a well-established body of evidence on the social disparities in caries distribution in children [23, 34, 35]. Children from worse-off families had significantly higher dmft scores than their peers from better-off families. Such disparities in caries experience can be explained by the effect of material deprivation on family environment and lifestyle leading to unfavourable behaviours among poorer families [23, 35, 36]. In the present study poor oral hygiene and near bedtime consumption of sugars were associated with higher caries rates. These results are in line with previous studies that explored caries risk factors in young children [19, 23, 37–40]. Although self-reported data must be interpreted with caution because of the potential social desirability bias [41], irregular tooth brushing and reduced consumption of sugars are highly expected observations in a setting of political crisis and civil wars [21, 25, 42]. However, further research is required to fully understand the pathways of social disparities in caries experience among Libyan children.

Some of the issues emerging from this study’s findings relate specifically to oral health planning and policies in Libya. Given that a concomitant increase in sugar consumption and caries incidence is highly expected in post-war environments [21], future oral health policies in Libya should aim to prevent caries and its related treatment needs as well as complications. The combination of the present study’s findings supports the use of caries risk assessment tools, based on social class and lifestyle behaviours, for caries prediction and prevention in dental practices [43]. Our findings, also, shed light on the need of oral health promotion programmes that focus on oral hygiene practices and healthy dietary habits, particularly among children from poorer families, in order to prevent dental caries and to reduce its related social disparities.

Another aim of the present study was to use a cross-culturally validated A-ECOHIS to assess OHRQoL impacts on Libyan children and their families. A_ECHOIS has been validated in Arabic culture and proved high level of validity and reliability. It can be seen that, although most of participants (70%) reported OHRQoL impacts, the mean scores of A-ECOHIS and its sections were lower than that observed in previous studies conducted in other countries [27]. Possible explanations for this could be cultural differences in the perception of oral heath impact or the fact that the study sample was recruited from asymptomatic population during vaccination campaign rather from dental patients who sought treatment [44].

In the present study, children from higher income families had lower OHRQoL impacts than their peers from lower socio-economic status. This finding agrees with the conclusion of a systematic review on the association between socio-economic status and OHRQoL among young children which suggests that socio-economic status indicators are significant predictors of OHRQoL [45]. On the other hand, children with history of dental caries (dmft ≥ 1) experienced significantly higher OHRQoL impacts than caries-free children in all domains and total scores of A-ECOHIS and its sections. These findings corroborate a great deal previous results reported in other countries such as Saudi Arabia [27], India [46], Brazil [44, 47] and China [48]. Oral health symptoms and related functions as well as parental distress appeared to be the most significantly affected aspects of A-ECOHIS among the Libyan children and their families. Therefore, the current study findings support the view that dental caries and socio-economic status, can predict OHRQoL in preschool children and highlights the need for assessing these impacts and their associated factors in future research.

Finally, a few important limitations need to be considered. First, the cross-sectional study design, although, useful for developing baseline data and for informing programme planning and research particularly in low resourced countries such as Libya [49], it does not allow for establishing any causal relationship [50]. Second, children with periodontal diseases were not excluded from the study and this may lead overestimation of caries impact on OHRQoL. However, periodontal problems were observed in very few cases and their effect can be neglected. Third, there is a potential risk of social desirability bias which is very likely when using self-administered questionnaires completed by parents as proxies for their children [41]. However, the respondents were asked to report their usual practices and assured that the study aimsto describe the actual practices and related variations rather than challenging their level of knowledge or judging their practices [51]. Finally, recall bias is another source of uncertainty which may possibly affect the validity of responses of the parent. However, parents are satisfactory proxies of young children who have limited cognitive abilities [52]. Future research using a prospective study design is, therefore, required to provide valid data on caries risk factors.

## Conclusions

The present study demonstrated high caries rates, unmet treatment needs and significant OHRQoL impacts among 6-years-old Libyan children. Low family income, near bedtime sugar intake and poor oral hygiene practices were associated with high caries rates. Dental caries and low family income negatively affected the OHRQoL of preschool children. Therefore, future preventive programmes should focus on promoting oral hygiene and healthy dietary habits, particularly among disadvantaged groups. Combining normative need assessment and socio-dental indicators in future assessment of oral health is required for programs planning and evaluation.

## Data Availability

The data sets used and/or analyzed during the current study are available from the corresponding author upon reasonable request.

## Abbreviations

OHRQoL: Oral health related quality of life
A-ECOHIS: Arabic Version of Early Childhood Oral Health Impact Scale
